# Caffeine citrate increases ciliary beat frequency in human respiratory epithelial cells

**DOI:** 10.1186/s40348-026-00239-y

**Published:** 2026-05-12

**Authors:** Sandra Cindrić, Laura Bodenbeck, Rim Hjeij, Niki Tomas Loges, Christine Edelbusch, Sebastian George, Petra Pennekamp, Esther Rieger-Fackeldey, Heymut Omran

**Affiliations:** 1 Department of General Paediatrics, Universitätsklinikum Münster, Albert-Schweitzer-Campus A1, Muenster, NRW 48149 Germany; 2 MVZ PränatGyn GmbH Mainz, Mainz, 55116 Germany; 3 Department of Paediatrics, Klinikum Rechts der Isar, Technische Universität München, Munich, Germany

**Keywords:** Caffeine citrate, Bronchopulmonary dysplasia, Neonates, Ciliary beat frequency, Air-liquid interface culture, Mucociliary clearance, Ryanodine receptor

## Abstract

**Background:**

Caffeine citrate is the gold-standard pharmacological treatment for apnea of prematurity, improving survival and long-term respiratory outcomes in preterm infants. Beyond its established central nervous system effects, emerging evidence suggests broader benefits in reducing extubation failure, lowering bronchopulmonary dysplasia rates, and decreasing the need for mechanical ventilation—but its direct actions on the respiratory epithelium remain largely unknown. Caffeine is a methylxanthine with multiple molecular targets, including adenosine receptor antagonism, phosphodiesterase inhibition, and stimulation of calcium (Ca²⁺) release from intracellular stores—mechanisms also linked to the regulation of ciliary beat frequency (CBF), a critical determinant of mucociliary clearance. Since impaired CBF is a hallmark of several chronic airway diseases, understanding whether caffeine citrate can directly modulate this function could broaden its therapeutic relevance. We hypothesized that caffeine citrate directly enhances CBF in human respiratory epithelial cells (hRECs), thereby supporting airway defense mechanisms in health and disease.

**Results:**

CBF was quantified by high-speed video microscopy in hRECs derived from healthy donors and individuals with cystic fibrosis (CF) cultured under air–liquid interface conditions. Caffeine citrate significantly increased CBF above baseline (CBF_b_) in both healthy and CF-derived cells, indicating a robust and reproducible stimulatory effect. Mechanistic experiments identified ryanodine receptor (RYR)–mediated Ca²⁺ efflux from intracellular stores as the key pathway, as pharmacological blockade of RYR abolished the caffeine-induced CBF enhancement.

**Conclusions:**

This study reveals a novel pharmacological effect of caffeine citrate—direct stimulation of CBF in primary hRECs through RYR-dependent Ca²⁺ signaling. By improving mucociliary clearance, caffeine citrate may offer peripheral airway benefits in addition to its central actions for apnea prevention. Given its favorable pharmacokinetic profile, long half-life, and good tolerability compared to other methylxanthines, caffeine citrate warrants further evaluation as a dual-action therapy for respiratory diseases with compromised clearance, including CF and bronchopulmonary dysplasia. These findings expand the mechanistic understanding of caffeine’s actions and highlight new translational opportunities in respiratory care.

**Supplementary Information:**

The online version contains supplementary material available at 10.1186/s40348-026-00239-y.

## Background

Methylxanthines are used for the therapy of a variety of respiratory disorders. Caffeine to treat neonates has been known for more than 60 years [1]. Since 2000, it is in use as citrate salt of caffeine for treatment of apnea of prematurity [2]. Newborns have special characteristics due to their physiological immaturity and their state of continuous development, resulting in medical conditions unique for this population. As consequence, a very small number of drugs are approved for neonates and data supporting the safety and efficacy are limited. Quite recently, considerable attention has been paid to clinical trials of caffeine citrate treatment for apnea of prematurity, the prevention of extubation failure and bronchopulmonary dysplasia, and the need for mechanical ventilation [3–10]. Current research focusses on effects on the central nervous system for apnea prevention.

Since caffeine citrate therapy for premature newborns is an effective therapy for several respiratory conditions, it is of great interest to investigate if caffeine citrate has also a direct function in the respiratory epithelium. Caffeine has several known mechanisms of actions: it is an adenosine receptor antagonist, releases calcium (Ca^2+^) from intracellular stores and inhibits phosphodiesterases [11]. Several of these pathways are known to be involved in regulation of the ciliary beating [12, 13]. These pathways include signalling via adenosine receptors, Ca^2+^ mediated events, phosphodiesterase, and guanylyl cyclase [14–17].

We hypothesized that caffeine citrate might constitute a potential pharmacological modulator for ciliary beat frequency (CBF) regulation in human respiratory cells. Therefore, we examined the effect of caffeine citrate on fully differentiated human primary respiratory epithelial cells (hRECs). Our results provide the first mechanistic evidence in hRECs that caffeine directly increases ciliary beat frequency (CBF) via ryanodine receptor (RYR)–dependent signaling. While this pathway appears to play a central role, our findings do not exclude the contribution of additional mechanisms, such as phosphodiesterase inhibition, modulation of GABA_A_​ receptors, or other signaling pathways. Despite this limitation, these data may help to explain why caffeine citrate has demonstrated clinical benefits not only in the prevention of apnea but also in reducing the incidence of bronchopulmonary dysplasia.

## Methods

### Cell isolation

Human respiratory epithelial cells were obtained by nasal brush biopsy from the middle turbinate of healthy donors (n = 5) and cystic fibrosis (CF) (n = 3) individuals. All reagents and collection media were equilibrated to room temperature prior to use. A sterile cell collection brush (Celletta™ brush cell collector with protective tip, product no. 9100060; Engelbrecht Medizin- und Labortechnik GmbH, Edermünde, Germany) was moistened with sterile isotonic saline solution. Equivalent sterile cytology brushes may be used. Before sampling, the participant was instructed to gently clean the nasal vestibule. The moistened brush was then introduced into the inferior nasal meatus and gently rubbed several times against the medial and superior aspects of the inferior turbinate using combined rotational and linear motions. This procedure typically induces transient local irritation and lacrimation, which subsides rapidly. Immediately after sampling, the brush was withdrawn and placed into the collection tube filled with 15 ml RPMI-1640 + HEPES (Invitrogen, Karlsruhe, Germany) and 2x Antibiotic-Antimycotic (100x Anti-Anti, Invitrogen, Karlsruhe, Germany). The brush was agitated vigorously within the tube at least 40 times to release the collected cells into the medium. The study was approved by the Ethics Committee of the Westphalian Wilhelms University of Muenster (2013-512-f-S). Written informed consent to participate in this study was obtained from each individual. Cells were resuspended in DMEM/Ham´s F12 (1:1) (Invitrogen, Karlsruhe, Germany) supplemented with 2% UltroserG (Pall Life Sciences, Germany) and 2x Antibiotic-Antimycotic and plated on collagen-coated tissue flasks. Type 1 collagen from rat tail tendon was isolated according to the protocol published by Rajan et al. [18] and diluted 1:5 with acetic acid prior to use. 4 ml of diluted rat-tail collagen in acetic acid (1:5) were placed into cell culture flasks and dried for 24 h at room temperature. Prior to use, collagen coated flasks were washed twice with 1xPBS (Invitrogen, Karlsruhe, Germany). Cells were cultured in a humidified atmosphere with 5% C2 at 37 °C. Medium was replaced three times per week until 70–80% cnfluence was reached. Ciliogenesis was induced in suspension culture or in air-liquid interface (ALI) culture.

### Cell culture of primary respiratory epithelial cells in suspension culture 

The collagen layer of cell culture flasks was resolved using collagenase type IV (200 U/mL; Worthington Biochemical Corporation, St. Katharinen, Germany) and the cell sheet was chopped up using a cell scraper. The cell suspension was washed in DMEM/Ham´s F12 (1:1) medium containing 1x Antibiotic-Antimycotic . The cell pellet was resuspended in 10 mL DMEM/Ham´s F12 (1:1) supplemented with 10% NU-Serum IV (Becton, Dickinson and Company) and transferred to an uncoated T25 cell culture flask. Cells were placed at an angle of 15° on a rotatory shaker and incubated at 37 °C. The medium was replaced three times a week. Between day 14 and 28 of suspension culture, spheroids were analyzed by high-speed video microscopy.

### Culture of primary respiratory epithelial cells at air-liquid interface (ALI) 

ALI inserts (Costar™ Corning^®^ 3470 Transwell™ Clear Polyester Membrane Inserts For 24-Well Plates, Corning Incorporated, USA) were collagen-coated using 250 µL diluted rat-tail collagen in acetic acid (1:5) per insert. After drying for 24 h at room temperature, inserts were washed twice with 1xPBS, before use. Cell layers of hRECs expanded on collagen-coated flasks were treated with collagenase type IV (200 U/mL; Worthington Biochemical Corporation, St. Katharinen, Germany) and cells were trypsinized for 5 min in 1x Trypsin-EDTA Solution (Sigma-Aldrich Co. LLC, St. Louis, USA). The cell pellet was resuspended in B-ALI Growth Basal Medium (Lonza, Cologne, Germany) and seeded into collagen coated transwell inserts (100,000 cells per insert). B-ALI Growth Basal and B-ALI differentiation media were purchased from Lonza as B-ALI™ BulletKit that included B-ALI™ Growth Basal Medium, B-ALI™ Differentiation Basal Medium (500 ml) and B-ALI™ SingleQuots Supplements. Supplements containing transferrin, B-ALI-Inducer, BPE (Bovine Pituitary Extract), epinephrine, GA-1000 (30 mg/ml gentamicin and 15 µg/ml amphotericin), hEGF, hydrocortisone, insulin, retinoic acid, and triiodothyronine were added to the growth and differentiation media as recommended by the manufacturer.

Cells were incubated with 5% CO2 at 37 °C in a humidified incubator and fed with B-ALI Growth Basal Medium from the basolateral and apical compartment every day. Once confluence was reached, air lift of the ALI culture was performed by aspirating the medium of the apical compartment and replacing medium of the basal compartment by B-ALI differentiation medium. Medium was replaced three times per week. Two to three inserts per individual were used for the analysis after a minimum of 30 days after air-lift and differentiation. This period ensures complete differentiation, which is considered achieved 21 days after air-lift.

### Video microscopy 

ALI cultures of hRECs ensure steady experimental settings for CBF measurements over time. CBF was measured in areas with active ciliation and restricted to a single field of view per insert (minimum 2 inserts/individual) to allow repeated short-interval recordings from the same location over several hours, as the study prioritised temporal reproducibility at a defined site over spatial heterogeneity.

Sisson-Ammons Video Analysis (SAVA) (Ammons Engineering, Mt Morris, MI, USA) was used for CBF analyses [19]. Videos of hRECs ALI were recorded using a Megaplus camera model ES 310 turbo (Redlake Inc., USA) attached to an inverted non-motorized phase-contrast microscope (Nikon Eclipse Ti-S) equipped with an ELWD 40x S Plan Fluor objective. Digital image sampling was performed at 125 fps and 640 × 480-pixel resolution for two seconds each (corresponding to 250 frames in total per measurement) over a period of 130–160 min without additional CO2 application. The Image focus was set and/or corrected if needed manually by the observer. The average interval between recordings of the same insert over the period of 130–160 min was 58 s. All experiments were performed under physiological conditions, by maintaining the temperature in the cell culture medium at 36 °C by a Minitube SC300 heating system (Minitube International AG).

The ciliary beat frequency over baseline (CBF_b_) was determined over a time period of 20 min. The individual measurements were fitted by Gaussian curves and their means were averaged to obtain the CBF_b_. CBF was measured over time, while treating the cells with defined drug concentrations. Pilot experiments demonstrated, that each application produced a brief transient decrease in CBF (< 1 min), followed by a sustained dose-dependent increase reaching a plateau after 15–20 min and persisting for ~ 30 min. This timeframe of 50 min was then used in follow up experiments for each drug dose. To test the dose response of caffeine citrate induced CBF excitation, CBF was measured over time, while treating cells from healthy control individuals and CF individuals sequentially with prewarmed (37 °C) 1 mM, 5 mM and 10 mM caffeine citrate in 15,6 µl total volume per insert (Peyona^®^, solution for infusion and oral solution), applied to the apical compartment of the insert. As control, 1mM and 5 mM citrate buffer were used. Sodium citrate dihydrate (Sigma-Aldrich) was dissolved in HPLC-grade water to prepare a 1 M stock solution (14.71 g in 50 mL), and the pH was adjusted to 7.5. The stock solution was subsequently diluted in cell culture medium as required.

**Fig. 1:**
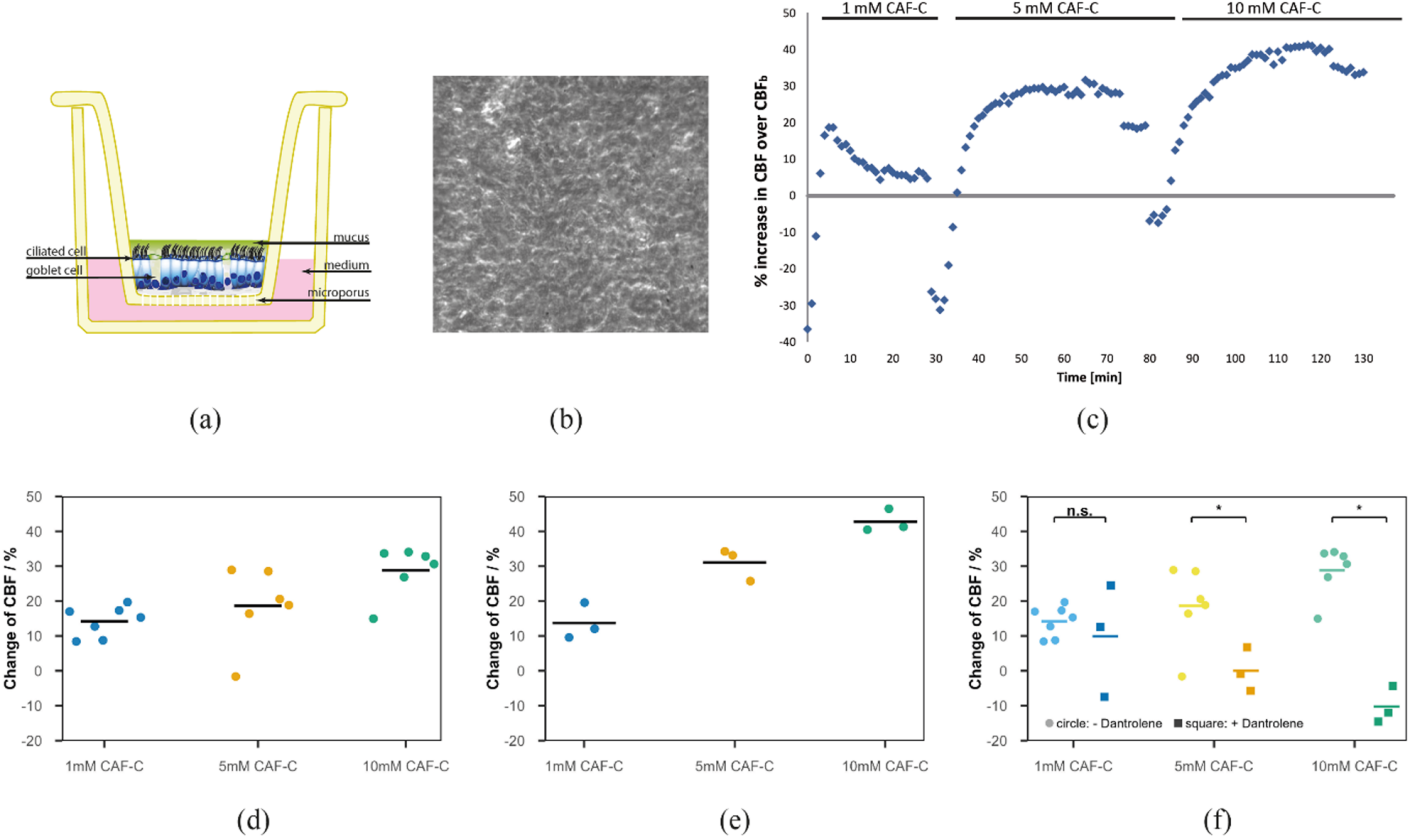
Caffeine citrate induced CBF increase via RYR channels. (**a**) schematic and (**b**) overview of human respiratory cells (hRECs) cultured at air-liquid interface (ALI). Tissue culture of fully differentiated hRECs at ALI conditions allow repeated CBF measurements of a large specific active area over time. (**c**-**e**) Administration of caffeine citrate into the apical compartment of the ALI transwell filters in increasing concentrations from 1 mM, 5 mM, to 10 mM results in a concentration-dependent increase of CBF over CBF_b_ in hRECs cultured at ALI in both hRECs of healthy donors (c, d. *n* = 7 (1 mM CaF-C), *n* = 6 (5 mM Caf-C and 10 mM CaF-C)) and in hRECs with deficient MCC obtained from CF individuals (e, *n* = 3). (**f**) Dantrolene inhibits the effect of caffeine-induced CBF excitation on hRECs (1 mM : p:0.37 (n.s.), 5 mM : *p* = 0.04 (*), 10 mM: *p* = 0.01 (*))

For application of dantrolene to healthy control cells, a stock solution of 10 mmol/l (3,362 mg/ml) dantrolene (Sigma, dantrolene sodium salt) was prepared dimethylformamide (DMF, Sigma). Final experimental concentration of dantrolene was 10µmol/ml within the basal cell culture medium (B-ALI differentiation medium). After CBF_b_ was measured for 30 min, dantrolene was applied to the basolateral compartment. This was followed by sequential addition of 1, 5, and 10 mM caffeine citrate to the apical compartment. CBF measurements were performed at each concentration for 30 min at 36 °C. For data presented in Fig. 1d-f, for each donor CBF frequency spectra within a time span of 20 min after reaching the peak plateau were used for calculation. Each spectrum was fitted by a Gaussian curve. The means of all Gaussian curves were averaged to derive the final value of that donor.

### Statistical analysis 

The obtained maximal increase in CBF from CBF_b_ in percent was compared between standard dose effect curves of caffeine citrate. Statistical analysis was performed using the SPSS software (www.ibm.com). Comparison was performed using the Wilcoxon signed-rank test. Significance was assumed at *P* < 0.05.

## Results

### Caffeine citrate increases CBF in hRECs and shows a dose-dependent effect

 Application of caffeine citrate to the healthy control spheroids in suspension culture showed a short-term decrease followed by a sustained increase in CBF over CBF_b_ in the presence of caffeine citrate (Supplementary Fig. 1). However, due to free movement of spheroids in suspension culture, it was impossible to measure CBF in the same area and in short time intervals, making it difficult to interpret the result. As consequence, we switched to ALI cultures of healthy controls and CF individuals to assess the impact of caffeine citrate on CBF.

Application of caffeine citrate to the apical compartment of the ALI culture (Fig. 1a and b) showed a short-term decrease followed by a sustained increase in CBF over CBF_b_ in the presence of caffeine citrate (Fig. 1c). In Fig. 1c, data points represent consecutive measurements of CBF for a single field of view of a single insert. We could assign the observed effect to caffeine, by ruling out any effect of citrate buffer only on CBF_b_ (*n* = 3, Supplementary Fig. 2). We demonstrate a dose-dependent effect on hRECs by increasing exposure to 1, 5, and 10 mM caffeine citrate, resulting on average in highly significant CBF excitation of 14.2% (*n* = 7; *p* = 0.009), 18.6% (*n* = 6; *p* = 0.023), and 28.8%(*n* = 6; *p* = 0.014) over CBF_b_ respectively (Fig. 1d).

### In *vitro* caffeine citrate application increases CBF in CFTR-mutant hRECs

To demonstrate that CBF can be also increased in hRECs with deficient mucociliary clearance capacity (MCC), we used hRECs cultured from three cystic fibrosis (CF) individuals with deleterious mutations in the cystic fibrosis transmembrane conductance regulator gene (*CFTR*) (OS-277: c.1521_1523delCTT (p.Phe508del)/c.1007T > A (p.Ile336Lys) compound heterozygous; OS-271: c.1521_1523delCTT (p.Phe508del) homozygous) and OS-288: c.1521_1523delCTT (p.Phe508del)/ c.3849 + 10kbC-> T compound heterozygous). As already demonstrated on cell cultures of hRECs of healthy donors (Fig. 1d), increasing concentrations of 1 mM, 5 mM, and 10 mM caffeine citrate result in CBF excitation of 13.7 (*n* = 3), 31.0 (*n* = 3), and 41.7 (*n* = 3) over CBF_b_ also in hRECs obtained from CF individuals (Fig. 1e). Though CBF excitation is highly analogical to the healthy controls, a thorough statistical analysis could not be performed due to the limited number of CF individuals analyzed.

### Caffeine citrate acts through RYR to modulate CBF 

**Fig. 2:**
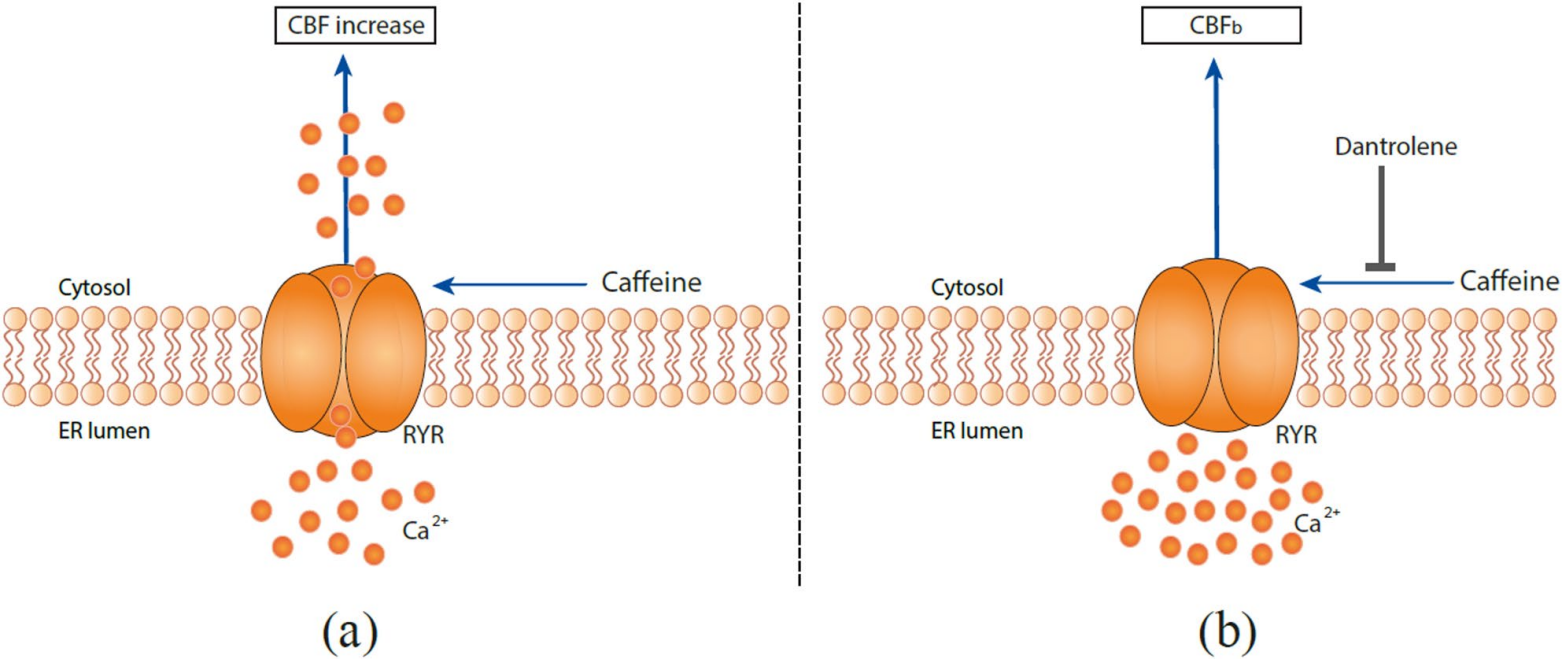
Proposed model of regulation of ciliary beating in human respiratory cells with regard to the effect of the pharmacological modulator caffeine and RYR channel blocker dantrolene. **a** Caffeine activates ryanodine receptors (RYR), promoting Ca^2+^ release from intracellular stores (endoplasmic reticulum, ER), which increases intracellular Ca^2+^ levels and stimulates ciliary beat frequency (CBF). **b** In the presence of dantrolene, RYR-mediated Ca^2+^ release is inhibited, thereby preventing caffeine-induced intracellular Ca^2+^ mobilization and abolishing the stimulatory effect of caffeine on CBF. Thus, dantrolene blocks the effect of caffeine by inhibiting the downstream Ca^2+^ release mediated through RYR. Abbreviations: ER = endoplasmic reticulum; RYR = ryanodine receptor

To determine the mechanisms of caffeine in CBF regulation, we performed additional pharmacological interventions. Caffeine is a known adenosine receptor antagonist, it releases Ca^2+^ from intracellular stores, and inhibits phosphodiesterases, all of which can modulate CBF [11]. In order to study Ca^2+^-mediated events on the basis of intracellular Ca^2+^ sources, Ca^2+^ signaling pathways involved in CBF regulation via RYR channels were investigated using the cell permeable RYR channel blocker dantrolene [20]. After CBF_b_ was measured for 30 min, dantrolene was applied to the basolateral compartment, followed by sequential addition of 1, 5, and 10 mM caffeine citrate to the apical compartment and CBF measurements at each concentration for 30 min. Here, dantrolene was found to inhibit the effect of caffeine-induced CBF excitation on hRECs (Figs. 1f and 2) (1 mM : p:0.37 (n.s.), 5 mM : *p* = 0.04 (*), 10 mM: *p* = 0.01 (*)). A concentration of 10 mmol/l dantrolene applied to the basolateral compartment of the hREC transwell insert results in significant inhibition of the effect of caffeine induced CBF-increase, resulting in an average reduction of CBF over CBF_b_ down to -10% (*n* = 3; 10 mM caffeine).

## Discussion

Our findings indicate that caffeine mediates CBF excitation via RYR channels in the human respiratory epithelium (Figs. 1f and 2), consistent with previous observations in the ciliated giant orange sea slug *Tritonia diomedea*. In this model organism, Woodward et al. demonstrated an inhibition of caffeine induced increase of CBF by dantrolene and concluded that caffeine is acting specifically through RYR channels to release Ca^2+^ from intracellular sources [12]. Thus, we demonstrate the evolutionary conservation of the functional role of caffeine in regulating CBF from gastropods, as previously demonstrated in the sea slug *Tritonia diomedea*, to mammals (humans).

In our study, we used sequential dosing the same culture. Each application produced a brief transient decrease in CBF (< 1 min), followed by a sustained dose-dependent increase reaching a plateau after 15–20 min and persisting for ~ 30 min. The short duration of the initial dip compared with the prolonged stimulatory response, together with the clear dose dependence, suggests that the transient decrease reflects acute physical or ionic perturbation during apical addition rather than cumulative pharmacologic effects. Therefore, sequential dosing is unlikely to affect the overall conclusion that caffeine citrate increases CBF.

It has to be noted, that caffeine citrate behaves differently in cell culture compared with neonates because only the latter involves whole-body pharmacokinetics. In cell culture, caffeine citrate is added directly to the medium, so there is no absorption phase and cells are exposed immediately to a stable, externally controlled concentration from the start of the experiment. This results in relatively constant exposure conditions over time. In contrast, in neonates, where caffeine citrate is given intravenously or orally with nearly complete bioavailability, the drug undergoes systemic distribution and elimination, leading to time dependent concentration profiles. Due to immature hepatic enzyme activity (especially CYP1A2), metabolism is limited, resulting in a prolonged half-live of roughly 65–100 h (far longer than in adults) and sustained systemic exposure that supports once-daily dosing.

These differences are important when interpreting our findings, as the cellular responses observed in vitro reflect exposure to constant concentrations, which may not fully replicate the dynamic pharmacokinetic profile in neonates. In particular, peak and trough levels, tissue distribution, and protein binding in vivo may influence both the magnitude and timing of drug effects. Therefore, our results should be understood as demonstrating cellular effects under controlled exposure conditions rather than directly predicting in vivo responses. Future studies incorporating time-varying exposure models or pharmacokinetic-informed experimental designs may help to better bridge this gap.

Although caffeine citrate increased ciliary beat frequency similarly in CF and healthy cells, the underlying cellular mechanisms mediating this response may differ. Because CFTR-mediated chloride transport is defective, caffeine-induced Ca²⁺ release may preferentially activate alternative Ca²⁺-dependent chloride channels, potentially partially compensating for impaired ion transport. In healthy cells, where CFTR is functional, this pathway may be less prominent. Additionally, caffeine’s antagonism of adenosine receptors could modulate signaling and ion transport differently in CF due to the altered cellular environment. However, based on our data, the effect of caffeine citrate on CBF was comparable in CF and healthy control individuals, despite potentially distinct underlying mechanisms.

Despite the fact that the experiments were conducted in human cell culture systems, which do not fully capture the complexity of in vivo conditions (including systemic interactions, metabolism, and tissue-level responses), and that only a single mechanistic pathway was investigated even though the drug may act via multiple biological routes, our findings remain promising and warrant further investigation in more complex models, including in vivo systems and across a broader range of respiratory diseases.

Caffeine citrate therapy has been shown to reduce the risk for bronchopulmonary dysplasia in neonates. Increased mucus production as well as mucus plugging play a role in the pathophysiology of bronchopulmonary dysplasia [21]. Our data indicate that the beneficial effect of caffeine citrate to reduce frequency of bronchopulmonary dysplasia in preterm infants might also result from increased CBF and improved cleaning of airways as mucus propelling velocity and thereby efficiency of MCC is linearly dependent on CBF [22].

In vitro, 1 mM caffeine citrate already induces a significant increase in CBF. Preterm infants treated for apnea of prematurity generally attain plasma caffeine concentrations in the range of approximately 0.015–0.103 mM, with therapeutic effects seen reliably above ~ 0.026 mM and occasionally episodes of tachycardia with higher plasma levels [23]. However, this discrepancy is more likely explained by differences in exposure dynamics and the absence of physiological modulators in vitro, including endogenous adenosine and other context-dependent factors that influence caffeine’s effects in vivo. To determine the clinical relevance of our findings, further studies assessing CBF before and after caffeine citrate administration in vivo will be necessary to evaluate whether therapeutically achievable concentrations can produce comparable effects in preterm infants.

We also propose that this direct function of caffeine citrate on the respiratory epithelium may be a promising approach to treat mucociliary clearance disorders as asthma (MIM 600807), chronic obstructive pulmonary disease (COPD; MIM 606963), or cystic fibrosis (CF; MIM 219700). Mucociliary dysfunction can result in substantial morbidity characterized by upper and lower respiratory tract infections, dyspnea, and chronic cough [24, 25]. Persistent infections and immune response can result in damage of the airways, bronchiectasis and chronic lung failure [26]. In consequence, improving MCC by increasing the CBF presents a promising strategy to treat lung diseases.

## Conclusion

The direct action of caffeine citrate on airway cells, together with its excellent bioavailability and licensed application even in premature infants, demonstrate great potential of methylxanthines for further research in this field. Various methylxanthines such as caffeine, theophylline, theobromine, aminophylline, or synthetic xanthines are already commonly used in respiratory disorders [11]. To emphasize, caffeine citrate has certain advantages over other methylxanthines, as it was shown to have a good performance relating to stable plasma concentration and longer half-life, and is better tolerated than e.g. theophylline [27]. Based on our findings, we conclude that caffeine citrate therapy for respiratory diseases with compromised mucociliary clearance should further be investigated.

## Supplementary Information

Supplementary Material 1.

## Data Availability

All data generated or analysed during this study are included in this article. Further enquiries can be directed to the corresponding author.

## Abbreviations

ALI: air-liquid interface
Ca²⁺: Calcium
CBF: Ciliary beat frequency
CBF_b_: CBF above baseline
CF: Cystic Fibrosis
COPD: chronic obstructive pulmonary disease
hRECs: Human respiratory epithelial cells
MCC: mucociliary clearance capacity
mM: millimolar
RYR: ryanodine receptor
SAVA: Sisson-Ammons Video Analysis
