# Supplementary material for: Caffeine citrate increases ciliary beat frequency in human respiratory epithelial cells
Source: Mol Cell Pediatr. 2026 May 12;13:27. doi: 10.1186/s40348-026-00239-y (PMC13168463; doi:10.1186/s40348-026-00239-y)
Supplement: Supplementary file 1 — Supplementary Material 1. [file 40348_2026_239_MOESM1_ESM.docx]

**Supplementary Figures**


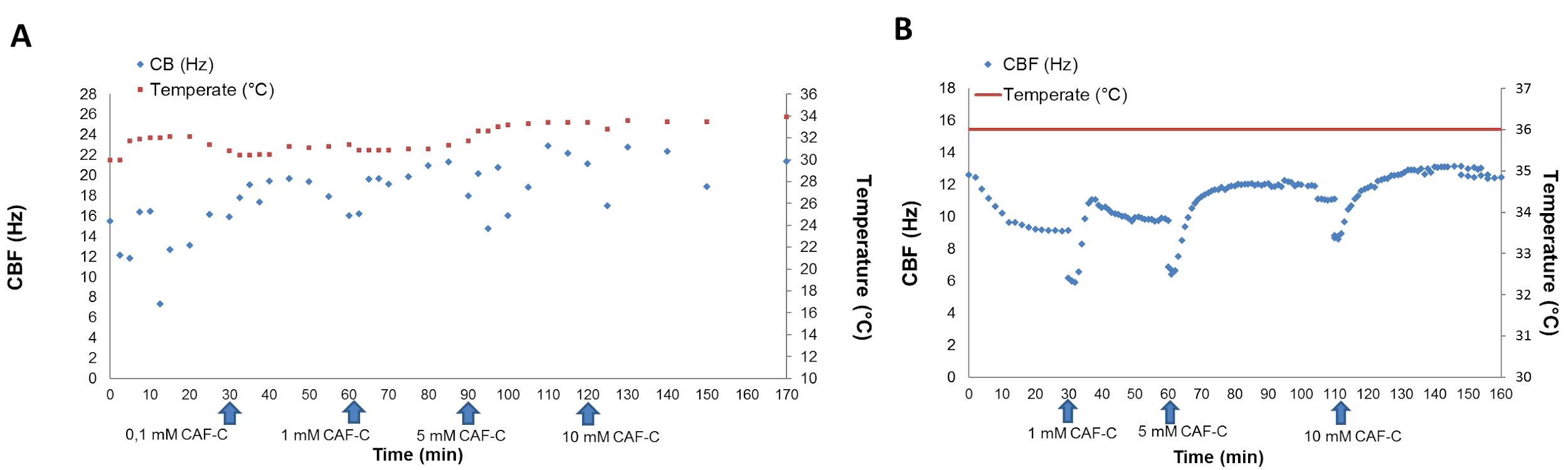


**Supplementary Figure 1:** Caffeine-induced increase of CBF in Human Respiratory Epithelial Cells (hRECs) in Suspension and ALI Cultures. (A) Increasing caffeine concentrations result in a rise of CBF in treated spheroids over base levels. (B) Results obtained by ALI based CBF measurements showed a concentration-dependent effect of 1 mM to 10 mM caffeine citrate (CAF-C) on CBF over CBF_b_. A visualization of the caffeine response of hRECs with a high temporal accuracy is given. The temperature is kept constant at 36 °C.


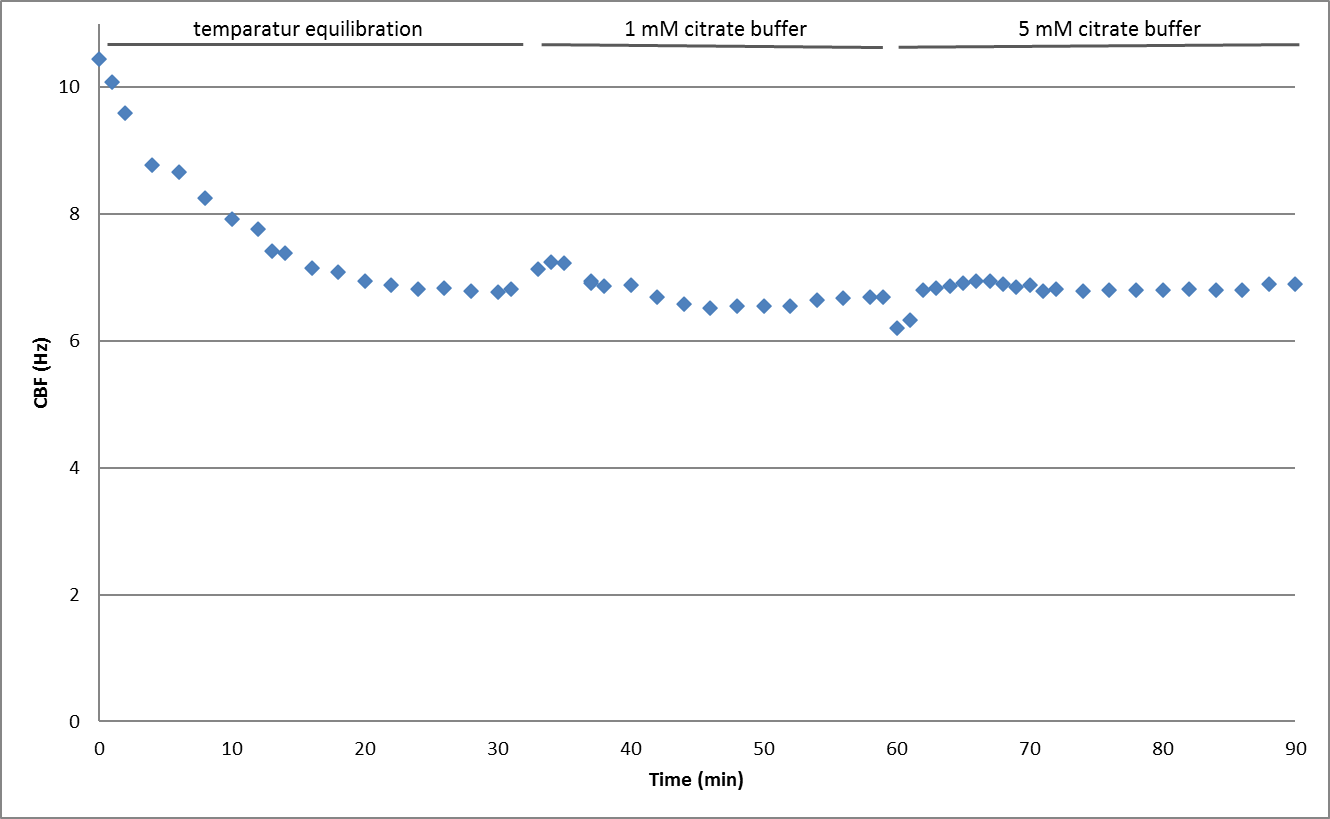


**Supplementary Figure 2:** Citrate buffer only has no impact on ciliary beat frequency (CBF). Neither one nor five mM citrate buffer increases CBF in human respiratory epithelial cells in air-liquid-interface (ALI) cultures. The temperature is kept constant at 36 °C.
